# Negentropy-Based Sparsity-Promoting Reconstruction with Fast Iterative Solution from Noisy Measurements

**DOI:** 10.3390/s20185384

**Published:** 2020-09-20

**Authors:** Yingxin Zhao, Yingjie Huang, Hong Wu, Ming Zhang, Zhiyang Liu, Shuxue Ding

**Affiliations:** 1 College of Electronic Information and Optical Engineering, Nankai University, Tianjin 300350, China; zhaoyx@nankai.edu.cn (Y.Z.); yjhuang97@mail.nankai.edu.cn (Y.H.); 2120170366@mail.nankai.edu.cn (M.Z.); liuzhiyang@nankai.edu.cn (Z.L.); sding@guet.edu.cn (S.D.); 2 Tianjin Key Laboratory of Optoelectronic Sensor and Sensing Network Technology, Tianjin 300350, China

**Keywords:** compressed sensing, sparse constraint, first order Taylor expansion, fast iterative, non-Gaussian noise

## Abstract

Compressed sensing provides an elegant framework for recovering sparse signals from compressed measurements. This paper addresses the problem of sparse signal reconstruction from compressed measurements that is more robust to complex, especially non-Gaussian noise, which arises in many applications. For this purpose, we present a method that exploits the maximum negentropy theory to promote the adaptability to noise. This problem is formalized as a constrained minimization problem, where the objective function is the negentropy of measurement error with sparse constraint $\ell_{p}\left(0<p<1\right)$-norm. On the minimization issue of the problem, although several promising algorithms have been proposed in the literature, they are very computationally demanding and thus cannot be used in many practical situations. To improve on this, we propose an efficient algorithm based on a fast iterative shrinkage-thresholding algorithm that can converge fast. Both the theoretical analysis and numerical experiments show the better accuracy and convergent rate of the proposed method.

## 1. Introduction

In recent years, compressed sensing (CS) has attracted considerable attention in areas of computer science, signal processing and wireless communication [1,2,3,4,5]. It can be considered as a signal sampling or coding approach that leads full use of signal sparsity, which may make it possible to surpass the traditional limits of sampling theory. CS suggests that if the signal is sparse or sparse in a transform domain such as the Fourier transform domain or wavelet transform domain, it can be projected onto a low-dimensional space via a measurement matrix that is uncorrelated with the transform basis. The original signal can be represented using only a few non-zero coefficients in a suitable basis or dictionary. Nonlinear optimization can then enable reconstruction of such signals from very few measurements with high probability. This reconstruction process can also be called sparse coding, which is described mathematically as finding an unknown signal α to satisfy the equation

(1) x=Dα+n

where $D\in {\mathbb{R}}^{M\times N}\left(M<N\right)$ is a measurement matrix that satisfies the restricted isometry property (RIP) criteria [6], $x\in {\mathbb{R}}^{M}$ is a low-dimensional measurement signal, $n\in {\mathbb{R}}^{M}$ is an unknown noise vector and $\alpha \in {\mathbb{R}}^{N}$ is a sparse signal or a sparse representation of the signal in a transform basis.

Obviously, this is solving an underdetermined equation, and there are infinite solutions. Given the measurements x and the knowledge that the original signal α is sparse or compressible, it is natural to attempt to recover α by treating this feature as a constraint [7]. Then, we can search for an optimal one from infinite solutions by solving an optimization problem of the form

(2) $$\underset{\alpha }{\min}||\alpha |{|}_{0}\;\mathrm{subject}\;\mathrm{to}\;x=D\alpha +n$$

where $||\alpha |{|}_{0}$, formally called $\ell_{0}$ regularization, counts the number of non-zero complements of α and is used to promote the sparsity of the solution [8]. However, if we want to solve the above problem directly, we need to list the combinations of all non-zero element positions separately. This is an NP-hard problem, which is computation prohibited [9]. In order to overcome the difficulty, many researchers are engaging in developing simple and effective algorithms to solve the $\ell_{0}$ regularization problem.

A typical method is using a greedy algorithm such as orthogonal matching pursuit (OMP) [10], compressive sampling matching pursuit (CoSaMP) [11], regularized orthogonal matching pursuit (ROMP) [12] and so on. The greedy strategies rely on iterative approximation of the signal coefficients and support, which are very fast for low-dimensional problems. Another approach is to relax $\ell_{0}$ regularization and, instead, to consider the $\ell_{1}$ regularization:

(3) $$\underset{\alpha }{\min}||\alpha |{|}_{1}\;\mathrm{subject}\;\mathrm{to}\;x=D\alpha +n$$

where $||\alpha |{|}_{1}$ is $\ell_{1}$ norm counting the sum of the absolute values of non-zero elements in vector α. Thus, the $\ell_{0}$ regularization problem is replaced by a more tractable formulation. That is, $||\cdot |{|}_{0}$ is replaced with its convex approximation $||\cdot |{|}_{1}$, whose minimization can be performed efficiently using conventional convex optimization methods. In fact, the resulting problem can be posed as the linear program. For problems (2) and (3), we can search unknown variable α using the method of gradient descent, which is called the iterative shrinkage-thresholding algorithm (ISTA) [13]. In addition, the basis pursuit (BP) algorithm [14] is also used for solving such problems.

There are a variety of algorithms that have been used for solving optimization problems in applications such as digital signal process and wireless communication. In these fields, noise inevitably affects the performance of the algorithm. The ability to perfectly reconstruct a sparse signal from noise-free measurements represents a very promising result. However, in most real-world systems the measurements are likely to be contaminated by some form of noise. Moreover, systems which are implemented in physical hard-ware will be subject to a variety of different types of noise depending on the setting [15].

In most previous studies, the constraint in (2) or (3) is formulated as a penalty function with the mean square error (MSE),

(4) $$\underset{\alpha }{\min}\{f\left(\alpha \right)=||x-D\alpha |{|}_{2}^{2}+\lambda ||\alpha |{|}_{1}\}$$

so that the problem becomes an unconstrained optimization problem and can be solved using a convex optimization algorithm. Here, MSE is adopted since it is optimal if the noise, n, is Gaussian distributed, which is indeed a suitable assumption for many applications.

However, there are many other applications in which the noise may not be Gaussian distributed. In these cases, if we still use the MSE measurement, it results in performance degrading and thus becomes not applicable in the targeted applications. In fact, non-Gaussian noise is quite common, such as the salt and pepper noise in image [16] and impulse noise in underwater acoustic channels [17]. Therefore, it is necessary to develop new methods and algorithms that are applicable to tasks with non-Gaussian noise.

### 1.1. Related Works

In order to achieve robust sparse recovery in the presence of non-Gaussian noise, various robust formulations have been proposed in recent years. The Lorentzian norm was employed as metrics for the residual error in [18]. In [19,20], optimization-based approaches using the Huber function as the objective function were proposed. Moreover, to tackle the $\ell_{1}$-regularized least-absolute ($\ell_{1}$-LA) minimization problem, many effective algorithms have been proposed such as YALL1 [21], which solves the $\ell_{1}$-LA formulation based on the alternating direction method of multipliers (ADMM) framework. Though $\ell_{1}$-norm regularization has been widely used, $\ell_{p}(0<p<1)$-norm regularization has shown significantly better performance in sparse recovery. To achieve more accurate recovery, research focusing on the $\ell_{p}$-LA minimization problem was reported in [22]. Meanwhile, it was demonstrated in [23] that the negentropy of measurement error can achieve good recovery performance with non-Gaussian noise. Whilst the focus of [23] is to present a novel method of error measurement, it also suggests an optimized algorithm that combines forward-backward splitting to solve the robust CS formulation. However, the efficiency and capacity of the method proposed in [23] have not reached the requirement of real-world applications that require fast processing, such as magnetic resonance imaging (MRI), thus, we propose in this paper more computationally efficient algorithms to achieve the goal.

### 1.2. Contributions

In this paper, we mainly study sparse signal reconstruction algorithms against non-Gaussian noise. A novel sparse representation model is proposed, which takes the negentropy [23] of measurement error as the objective function and $\ell_{p}\left(0<p<1\right)$ norm as the sparsity measurement. The negentropy can be exploited to measure the non-Gaussian properties of random variables, here the fitting error, and the stronger the non-Gaussian noise is, the larger the negentropy value is. Thus, this model is appropriate for reconstructing sparse signals from non-Gaussian noisy measurements, and α, which maximizes the negentropy of the measurement error, is the optimal solution if the constraint is satisfied. In summary, the contribution of this paper is as follows:

(a) We propose a new sparse representation model for accurately recovering the original signal from noisy measurements, especially if it is non-Gaussian. The negentropy of measurement error emerges as the loss function with optimum $\ell_{p}\left(0<p<1\right)$ sparse constraint.
(b) We develop a solution for the proposed model. The first order Taylor formula is used to approximate the value in the neighborhood at any point on the objective function, and then, we find the minimum point of the function in the neighborhood.
(c) We develop an optimization method of the fast iterative shrinkage-thresholding algorithm (FISTA) to speed up the convergence of the algorithm.
(d) We evaluated the new algorithm using sparse signal recovery, image denoising and recovery of MRI images. The results showed that the new algorithm is more accurate and faster than several existing methods and has better adaptability to different types of noise.

### 1.3. Organization

The rest of this paper is organized as follows. In Section 2, we survey the iterative shrinkage-thresholding algorithm (ISTA), since this technique will be used for the new model proposed in this paper. Then, we propose the algorithm model based on negentropy maximization and $\ell_{p}\left(0<p<1\right)$ norm and demonstrate the solution process of the model and the principle of accelerating convergence in detail. In Section 3, we show the experiments with a series of recovering sparse signals in different conditions to analyze the accuracy and stability of the developed algorithm. Furthermore, we show the performance of the algorithm in image denoising and MRI images recovery to indicate the performance in applications. Finally, Section 4 concludes the paper.

## 2. Methods

The problem that needs to be solved in this paper is to recover the sparse signal with high precision from the linearly compressed measurements with different types of noise, which is formulated by (1). Here, $\alpha \in {\mathbb{R}}^{N}$ is an unknown sparse signal in which the number k of non-zero element is less than N. $D\in {\mathbb{R}}^{M\times N}$ is a known measurement matrix with M<N, where the ratio of M to N is called the compression ratio. The measurement signal $x\in {\mathbb{R}}^{N}$, as a linear combination of column vectors in the D, is also known. The vector n may be Gaussian or non-Gaussian noise. In order to find the optimal solution, it is very necessary to establish a suitable mathematical model, which will play an important role in guaranteeing the accuracy of the solution.

### 2.1. ISTA Algorithm

ISTA is a typical algorithm used for solving problem (3); the detailed steps are shown in Algorithm 1, which can be frequently transformed into the following problem:

(5) $$\underset{\alpha }{\min}\{f\left(\alpha \right)=||x-D\alpha |{|}_{2}^{2}+\lambda ||\alpha |{|}_{1}\}$$

where $||\alpha |{|}_{1}=\sum_{i}^{n}\left|\alpha_{i}\right|$ is a sparse constrain term and *N* is the count of elements of signal α. λ>0 is a regularization parameter to adjust the effect of the sparse constraint. ISTA gradually approaches the global minimum after multiple iterations by finding the local minimum, which approaches the optimal solution according to following recursive equation [24]:

(6) $$\alpha_{k}=\;\pi_{\lambda \eta }\left(\alpha_{k-1}-\eta \nabla f\left(\alpha_{k-1}\right)\right)$$

where $\eta \in \left(0,1/||D^{T}D||\right)$ is a suitable step and $\pi_{\lambda \eta }$ is a nonlinear soft-thresholding operator, which is defined in Equation (7).

(7) $$\pi_{\lambda \eta }{\left(\alpha \right)}_{i}=\mathrm{sign}\left(\alpha_{i}\right)\max\{\left(|\alpha_{i}|-\lambda \eta \right),0\}$$

ISTA has been developed for a long time and is used in applications, but its application range is limited due to its poor performance against non-Gaussian noise. At the same time, the effect of $\ell_{1}$ norm as sparse constraint needs to be improved. Therefore, we aim, in this paper, to improve the robustness and accuracy of the algorithm by further changing the solution model.
**Algorithm 1** ISTA1: Input: measurement matrix D, measurement signal x.2: Initialization: $\eta \in \left(0,1/||D^{T}D||\right)$, $\lambda >0,\;\alpha_{0}=0,\;k=0$
3: Main iteration: increment k by 14:
$\alpha_{k}=$
$\pi_{\lambda \eta }$
$\left(\alpha_{k-1}-\eta \nabla f\left(\alpha_{k-1}\right)\right)$
5: Stopping rule: stop if $\alpha_{k}$ has converged6: Output: sparse signal α


### 2.2. Negentropy-Based and Sparsity-Promoting Algorithm

We propose to exploit the negentropy of measurement error, e=x−Dα, as the objective function as a new model. According to information theory, among all random variables with equal variance, the entropy of Gaussian variables is the largest [23]. Obviously, entropy can measure the Gaussian property of a variable, so negentropy can be used to measure its non-Gaussian property; the larger the negentropy is, the stronger the non-Gaussian variable is. Therefore, it is possible to find an optimal α which maximizes the negentropy of the measurement error so that an accurate estimation is obtained from the signal affected by the non-Gaussian noise. The negentropy is defined as follows:

(8) $$N\left(e_{i}\right)=H\left(e_{i_{gauss}}\right)-H\left(e_{i}\right),\;for\;i=1,2,\cdot \cdot \cdot ,M$$

where $e_{i_{gauss}}$ is a Gaussian distributed variable which has the same variance as $e_{i}$ and H(⋅) denotes the differential entropy of the random variable. It is worthy of notice that $N\left(e_{i}\right)$ is positive-definite. It is known from the negentropy definition that when $e_{i}$ follows the Gaussian distribution, $N\left(e_{i}\right)$ is zero. As $e_{i}$ becomes more non-Gaussian, $N\left(e_{i}\right)$ becomes larger. Because it is difficult to solve the differential entropy of random variables, the approximate expression (9) of negentropy is commonly used in practical applications.

(9) $$N\left(e\right)={\left\{E[G(e_{i})]-E[G(e_{i_{gauss}})]\right\}}^{2}$$

where G(⋅) is a non-linear function, such as tanh(c⋅e), $e\cdot \exp(-e^{2}/2)$ and $e^{3}$. E(⋅) is the expected value operation. Since $E[G(e_{i})]$ is less than or equal to $E[G(e_{i_{gauss}})]$, maximizing N(e) can be converted to minimize E[G(e)] and written as

(10) $$\underset{\alpha }{\min}\{f(\alpha )\}=\sum_{i=1}^{M}\left\{E[G(e_{i})]\right\}=\sum_{i=1}^{M}\left\{E[G{\left(x-D\alpha \right)}_{i}]\right\},\alpha \in {\mathbb{R}}^{N}$$

To solve the above formulation, we find the local minimum to converge the objective function to the global optimal solution through multiple iterations. First, we approximate the function f(α) with the first-order Taylor formula near the initial point $\alpha_{0}$.

(11) $$f(\alpha )=f(\alpha_{0})+\langle \alpha -\alpha_{0},\nabla f(\alpha_{0})\rangle$$

where f(α) represents the function value in the neighborhood of point $\alpha_{0}$ and $\nabla f(\alpha_{0})$ is the gradient of function f(α) at this point. Then, we solve the minimum value point $\alpha_{k}$ of the function in this neighborhood and use this point as the starting point of the next iteration. After multiple iterations, $\alpha_{k}$ will converge to the global optimal solution $\alpha^{*}$, which approaches the optimal solution according to the following recursive equation:

(12) $$\alpha_{k}=\underset{\alpha }{\mathrm{argmin}}\{f(\alpha_{k-1})+\langle \alpha -\alpha_{k-1},\nabla f(\alpha_{k-1})\rangle \}$$

A surrogate function based on the proximal point algorithm mentioned in [25,26] can be defined. Then, the objective function f(α) is replaced by

(13) $$J(\alpha ,\alpha_{k-1})=f(\alpha_{k-1})+\langle \alpha -\alpha_{k-1},\nabla f(\alpha_{k-1})\rangle +\frac{1}{2\eta }||\alpha -\alpha_{k-1}|{|}_{2}^{2}$$

where $\eta \in \left(0,1/||D^{T}D||\right)$ is a restriction parameter. For simplification, the above formula can be written as

(14) $$J(\alpha ,\alpha_{k-1})=\frac{1}{2\eta }||\alpha -(\alpha_{k-1}-\eta \nabla f(\alpha_{k-1}))|{|}^{2}+K$$

where $K=f(\alpha_{k-1}+\eta /2\nabla f(\alpha_{k-1}))$, a constant term, can be ignored, so Equation (12) can be rewritten as

(15) $$\alpha_{k}=\underset{\alpha }{\mathrm{argmin}}\{\frac{1}{2\eta }||\alpha -(\alpha_{k-1}-\eta \nabla f(\alpha_{k-1}))|{|}^{2}\}$$

In this paper, log[cosh(c⋅e)] is used as the nonlinear function in the negentropy formula, so the gradient of the function f(α) is expressed as $\nabla f(\alpha )=D^{T}\tanh(c(D\alpha -x))$. Adopting the $\ell_{1}$ regularization as sparse constraint to ensure the sparsity of the solution, $\alpha_{k}$ can be gained iteratively as follows:

(16) $$\alpha_{k}=\underset{\alpha }{\mathrm{argmin}}\{\frac{1}{2\eta }||\alpha -(\alpha_{k-1}-\eta D^{T}\tanh(c(D\alpha_{k-1}-x)))|{|}_{2}^{2}+\lambda ||\alpha |{|}_{1}\}$$

Since the $\ell_{1}$ norm is separable, the computation of $\alpha_{k}$ reduces to solving a one-dimensional minimization problem for each of its components, which can be produced by calculating the following formula:

(17) $$\alpha_{i}^{k}=\mathrm{sign}(\beta_{i}^{k-1})\max(0,|\beta_{i}^{k-1}|-\lambda \eta )$$

where we use the fact that $\beta_{i}^{k-1}=\alpha_{i}^{k-1}-\eta \nabla f(\alpha_{i}^{k-1})$; the detailed steps based on $\ell_{1}$ norm are shown in Algorithm 2, which has the same sparse constraint and different objective function as ISTA, so that we can further illustrate the advantages of the proposed objective function by comparing the two methods.
**Algorithm 2** Negentropy + $\ell_{1}$ Norm1: Input: measurement matrix D, measurement signal x.2: Initialization: $\eta \in \left(0,1/||D^{T}D||\right)$, $\lambda >0,\;\alpha_{0}=0,\;k=0$
3: Main iteration: increment k by 14:
$\beta_{i}^{k-1}=\alpha_{i}^{k-1}-\eta \nabla f(\alpha_{i}^{k-1})$
5:
$\alpha_{i}^{k}=\mathrm{sign}(\beta_{i}^{k-1})\max(0,|\beta_{i}^{k-1}|-\lambda \eta )$
6: Stopping rule: stop if $\alpha_{k}$ has converged7: Output: sparse signal α


$\ell_{p}\left(0<p<1\right)$ regularization, a nonconvex and non-smooth optimization problem, has been proven to solve more accurate sparse solutions than $\ell_{1}$ regularization as a relaxation approach in [27,28,29]. Weighted $\ell_{1}$ regularization is an approximate representation of $\ell_{p}$ regularization [30,31]. Adopting the weighted $\ell_{1}$ regularization as sparse constraint, the iterative scheme can be written as follows:

(18) $$\begin{array}{ll} \alpha_{k} & =\underset{\alpha }{\mathrm{argmin}}\{\frac{1}{2\eta }||\alpha -(\alpha_{k-1}-\eta D^{T}\tanh(c(D\alpha_{k-1}-x)))|{|}_{2}^{2}+\lambda ||\alpha |{|}_{p}^{p}\}\\ & =\underset{\alpha }{\mathrm{argmin}}\underset{i}{\Sigma }\{\frac{1}{2\eta }||\alpha_{i}-(\alpha_{i}^{k-1}-\eta D^{T}\tanh(c(D\alpha_{k-1}-x)))|{|}_{2}^{2}+\lambda w_{i}^{k-1}|\alpha_{i}|\} \end{array}$$

where $\left||\alpha |{|}_{p}^{p}=\sum_{i=1}^{N}|\alpha_{i}{|}^{p}\approx \sum_{i=1}^{N}(|\alpha_{i}|+\delta {)}^{p-1}|\alpha_{i}\right|$ is an approximate representation of the $\ell_{p}$ norm, δ is close to 0 and $w_{i}^{k}={\left(|\alpha_{i}^{k-1}+\delta |\right)}^{p-1}$ is a constant. It is clear from (18) that $\alpha_{i}$ respectively in each dimension of vector $\alpha_{k}$ can be solved and updated by calculating the following formula:

(19) $$\alpha_{i}^{k}=\mathrm{sign}(\beta_{i}^{k-1})\max(0,|\beta_{i}^{k-1}|-\lambda \eta w_{i}^{k-1})$$

The detailed steps based on a weighted $\ell_{1}$ norm are shown in Algorithm 3, which improve accuracy of the solution.
**Algorithm 3** Negentropy + Weighted $\ell_{1}$ Norm1: Input: measurement matrix D, measurement signal x.2: Initialization: choose proper η, λ, c, p and δ, $\alpha_{0}=0,k=0$
3:
Main iteration: increment k by 14:
$\beta_{i}^{k-1}=\alpha_{i}^{k-1}-\eta \nabla f(\alpha_{i}^{k-1})$
5:
$\alpha_{i}^{k}=\mathrm{sign}(\beta_{i}^{k-1})\max(0,|\beta_{i}^{k-1}|-\lambda \eta w_{i}^{k-1})$
6: Stopping rule: stop if $\alpha_{k}$ has converged7: Output: sparse signal α


An exact solution can also be solved by analyzing the trend of the function. For $\ell_{p}$ regularization, there will be different sparse constraint effects with different p. When p∈(0,0.5], there is no significant difference, but when p∈[0.5,1), the smaller the value of p is, the better the sparse effect is [32,33]. Thus, $\ell_{p}$ norm, where p=0.5, is selected as sparse constrain in the proposed algorithm. Adopting $\ell_{0.5}$ regularization as sparse constraint, the iterative scheme can be written as

(20) $$\alpha_{k}=\underset{\alpha }{\mathrm{argmin}}\{\frac{1}{2\eta }||\alpha -(\alpha_{k-1}-\eta D^{T}\tanh(c(D\alpha_{k-1}-x)))|{|}_{2}^{2}+\lambda ||\alpha |{|}_{1/2}^{1/2}\}$$

According to the iterative half thresholding algorithm (IHTA) [34], this can be rewritten as

(21) $$\alpha_{i}^{k}=\frac{2}{3}x(1+\cos(\frac{2}{3}\cos^{-1}(-\frac{3^{3/2}}{4}\lambda \eta |x{|}^{-3/2})))\mathrm{sign}(\max(0,|\beta_{i}^{k-1}|-\frac{3}{2}\lambda \eta^{\frac{2}{3}}))$$

The detailed steps based on $\ell_{0.5}$ norm are shown in Algorithm 4.
**Algorithm 4** Negentropy + $\ell_{0.5}$ Norm1: Input: measurement matrix D, measurement signal x.2: Initialization: choose proper $\eta ,\;\lambda ,\;c,\alpha_{0}=0,\;k=0$
3: Main iteration: increment k by 14:
$\beta_{i}^{k-1}=\alpha_{i}^{k-1}-\eta \nabla f(\alpha_{i}^{k-1})$
5:
$\alpha_{i}^{k}=\frac{2}{3}x(1+\cos(\frac{2}{3}\cos^{-1}(-\frac{3^{3/2}}{4}\lambda \eta |x{|}^{-3/2})))\mathrm{sign}(\max(0,|\beta_{i}^{k-1}|-\frac{3}{2}\lambda \eta^{\frac{2}{3}}))$
6: Stopping rule: stop if $\alpha_{k}$ has converged7: Output: sparse signal α


### 2.3. Optimizing the Rate of Convergence

The proposed algorithm based on maximizing negentropy and $\ell_{p}$ regularization can reconstruct sparse signals more accurately against non-Gaussian noise, which has already been shown in the following experiment. Equally importantly, further optimization of the convergence rate is required since wide application of a CS algorithm, especially in processing large scale data [35], is decided by the optimization process for reconstruction.

In this paper, a fast way to find the optimal solution by optimizing the starting point in each iteration is presented, which is called the fast iterative shrinkage-thresholding algorithm (FISTA). We optimize the convergence rate of the proposed algorithm according to the way of selecting the starting point in FISTA. The detailed steps can be written as follows [24]:

(22) $$t_{k+1}=\frac{1+\sqrt{1+4t_{k}^{2}}}{2}$$

(23) $$\theta_{k+1}=\alpha_{k}+\left(\frac{t_{k}-1}{t_{k+1}}\right)(\alpha_{k}-\alpha_{k-1})$$

where $t_{1}=1$, $\theta_{k+1}$ depends on the linear combination of $\alpha_{k}$ and $\alpha_{k-1}$. After one iteration, we optimize the local minimum point as the input for the next iteration. ISTA has a worst-case complexity result of O(1/k), but FISTA has an improved complexity result of $O(1/k^{2})$. Our analysis also provides proof as follows.

Let F(α)=f(α)+g(α), $Q(\alpha ,\alpha_{n})=J(\alpha ,\alpha_{n})+g(\alpha )$, g(α) is a regularization function.

**Lemma 1.**  *Let*$\alpha_{n}\in {\mathbb{R}}^{N}$*and*η>0*be such that*

(24) $$F(\alpha_{n+1})\leq Q(\alpha_{n+1},\alpha_{n})$$

*Then for any*$\alpha \in {\mathbb{R}}^{N}$,

(25) $$F(\alpha )-F(\alpha_{n})\geq \frac{1}{2\eta }||\alpha_{n+1}-\alpha_{n}|{|}^{2}+\frac{1}{\eta }\langle \alpha_{n}-\alpha ,\alpha_{n+1}-\alpha_{n}\rangle$$

**Theorem 1.**  *Let*$\left\{\alpha_{k}\right\}$*be the sequence generated by* (6). *Then for any* k≥1

(26) $$F(\alpha_{k})-F(\alpha^{*})\leq \frac{||\alpha^{*}-\alpha_{0}|{|}^{2}}{2k\eta }\forall \alpha^{*}\in X_{*}$$

**Proof.**  Invoking Lemma 1 with $\alpha =\alpha^{*}$, we obtain

(27) $$\begin{array}{cc} 2\eta (F(\alpha^{*})-F(\alpha_{n+1})) & \geq ||\alpha_{n+1}-\alpha_{n}|{|}^{2}+2\langle \alpha_{n}-\alpha^{*},\alpha_{n+1}-\alpha_{n}\rangle \\ & =||\alpha^{*}-\alpha_{n+1}|{|}^{2}-||\alpha^{*}-\alpha_{n}|{|}^{2} \end{array}$$

Summing this inequality over n=0,⋅⋅⋅,k−1 gives

(28) $$2\eta (kF(\alpha^{*})-\sum_{n=0}^{k-1}F(\alpha_{n+1}))\geq ||\alpha^{*}-\alpha_{k}|{|}^{2}-||\alpha^{*}-\alpha_{0}|{|}^{2}$$

Invoking Lemma 1 one more time with $\alpha =\alpha_{n}$ yields

(29) $$2\eta (F(\alpha_{n})-F(\alpha_{n+1}))\geq ||\alpha_{n+1}-\alpha_{n}|{|}^{2}$$

Multiplying the last inequality by *n* and summing over n=0,⋅⋅⋅,k−1, we obtain

(30) $$2\eta \sum_{n=0}^{k-1}(nF(\alpha_{n})-(n+1)F(\alpha_{n+1})+F(\alpha_{n+1}))\geq \sum_{n=0}^{k-1}n||\alpha_{n+1}-\alpha_{n}|{|}^{2}$$

which simplifies to

(31) $$2\eta (-kF(\alpha_{k})+\sum_{n=0}^{k-1}F(\alpha_{n+1}))\geq \sum_{n=0}^{k-1}n||\alpha_{n+1}-\alpha_{n}|{|}^{2}$$

Adding (28) and (31), we obtain

(32) $$2k\eta (F(\alpha^{*})-F(\alpha_{k}))\geq ||\alpha^{*}-\alpha_{k}|{|}^{2}+\sum_{n=0}^{k-1}n||\alpha_{n+1}-\alpha_{n}|{|}^{2}-||\alpha^{*}-\alpha_{0}|{|}^{2}$$

and hence it follows that

(33) $$k\leq \frac{||\alpha^{*}-\alpha_{0}|{|}^{2}}{2\eta (F(\alpha_{k})-F(\alpha^{*}))}$$

The above result shows that when $\alpha_{k}$ converge to the optimal solution, the number of iterations is at most ${\left\|\alpha^{*}-\alpha_{0}\right\|}^{2}/2\eta (F(\alpha_{k})-F(\alpha^{*}))$. □

**Lemma 2.**  *The sequences*$\left\{\alpha_{k},\theta_{k}\right\}$*generated via FISTA with either a constant or backtracking stepsize the satisfy for every*k≥1

(34) $$2\eta (t_{k}^{2}v_{k}-t_{k+1}^{2}v_{k+1})\geq ||u_{k+1}|{|}^{2}-||u_{k}|{|}^{2}$$

where $v_{k}=F(\alpha_{k})-F(\alpha^{*})$.

**Lemma 3.**  *Let* $\left\{a_{k},b_{k}\right\}$ *be the position sequence of real satisfying*

(35) $$a_{k}-a_{k+1}>b_{k+1}-b_{k}\;\forall k\geq 1,\;with\;a_{1}+b_{1}\leq c,c>0$$

Then, $a_{k}<c$ for every k≥1.

**Lemma 4.**  *The positive sequence*$\left\{t_{k}\right\}$*generated in FISTA via* (2.3) *with* $t_{1}=1$ *satisfies* $t_{k}\geq (k+1)/2$ *for all* k≥1.

**Theorem 2.**  *Let* $\left\{a_{k}\},\{b_{k}\right\}$ *be generated by FISTA. Then, for any* k≥1

(36) $$F(\alpha_{k})-F(\alpha^{*})\leq \frac{||\alpha_{0}-\alpha^{*}|{|}^{2}}{2\eta {\left(k+1\right)}^{2}}\forall \alpha^{*}\in X_{*}$$

**Proof.**  Let us define the quantities

$$a_{k}=2\eta t_{k}^{2}v_{k},\;b_{k}=||u_{k}|{|}^{2},\;c=||\theta_{1}-\alpha^{*}|{|}^{2}-||\alpha_{0}-\alpha^{*}|{|}^{2}$$

and recall that $v_{k}=F(\alpha_{k})-F(\alpha^{*})$. Then, using Lemma 3, we have for every k≥1

$$a_{k}-a_{k+1}>b_{k+1}-b_{k}$$

and hence assuming that $a_{1}+b_{1}\leq c$ holds true, invoking Lemma 3, we obtain that

(37) $$2\eta t_{k}^{2}v_{k}\leq ||\alpha_{0}-\alpha^{*}|{|}^{2}$$

which, combined with $t_{k}\geq (k+1)/2$, yields. □

(38) $$k\leq \sqrt{\frac{||\alpha_{0}-\alpha^{*}|{|}^{2}}{2\eta (F(\alpha_{k})-F(\alpha^{*}))}}-1$$

It is important to note that, when $\alpha_{k}$ converge to the optimal solution, the number of iterations is at most $\sqrt{\;{\left\|\alpha^{*}-\alpha_{0}\right\|}^{2}/2\eta (F(\alpha_{k})-F(\alpha^{*}))}-1$. Obviously, FISTA has fewer iterations than ISTA. The optimized algorithms are shown separately in Algorithms 5–7.
**Algorithm 5** Fast Negentropy + $\ell_{1}$ Norm1: Input: measurement matrix D, measurement signal x.2: Initialization: $\eta \in \left(0,1/||D^{T}D||\right)$, $\lambda >0,\;\theta_{1}=\alpha_{0}=0,\;k=0,\;t_{1}=1$
3: Main iteration: increment k by 14:
$\beta_{i}^{k-1}=\theta_{i}^{k-1}-\eta \nabla f(\theta_{i}^{k-1})$
5:
$\alpha_{i}^{k}=\mathrm{sign}(\beta_{i}^{k-1})\max(0,|\beta_{i}^{k-1}|-\lambda \eta )$
6:
$t_{k+1}=\frac{1+\sqrt{1+4t_{k}^{2}}}{2}$
7:
$\theta_{k+1}=\alpha_{k}+\left(\frac{t_{k}-1}{t_{k+1}}\right)(\alpha_{k}-\alpha_{k-1})$
8: Stopping rule: stop if $\alpha_{k}$ has converged9: Output: sparse signal α


**Algorithm 6** Fast Negentropy + Weighted $\ell_{1}$ Norm1: Input: measurement matrix D, measurement signal x.2: Initialization: choose proper η, λ, c, p and δ, $\theta_{1}=\alpha_{0}=0,\;k=0,\;t_{1}=1$
3: Main iteration: increment k by 14:
$\beta_{i}^{k-1}=\theta_{i}^{k-1}-\eta \nabla f(\theta_{i}^{k-1})$
5:
$\alpha_{i}^{k}=\mathrm{sign}(\beta_{i}^{k-1})\max(0,|\beta_{i}^{k-1}|-\lambda \eta w_{i}^{k-1})$
6:
$t_{k+1}=\frac{1+\sqrt{1+4t_{k}^{2}}}{2}$
7:
$\theta_{k+1}=\alpha_{k}+\left(\frac{t_{k}-1}{t_{k+1}}\right)(\alpha_{k}-\alpha_{k-1})$
8: Stopping rule: stop if $\alpha_{k}$ has converged9: Output: sparse signal α


**Algorithm 7** Fast Negentropy + $\ell_{0.5}$ Norm1: Input: measurement matrix D, measurement signal x.2: Initialization: choose proper η, λ, c,3:
$\theta_{1}=\alpha_{0}=0,k=0,t_{1}=1$
4: Main iteration: increment k by 15:
$\beta_{i}^{k-1}=\theta_{i}^{k-1}-\eta \nabla f(\theta_{i}^{k-1})$
6:
$\alpha_{i}^{k}=\frac{2}{3}x(1+\cos(\frac{2}{3}\cos^{-1}(-\frac{3^{3/2}}{4}\lambda \eta |x{|}^{-3/2})))\mathrm{sign}(\max(0,|\beta_{i}^{k-1}|-\frac{3}{2}\lambda \eta^{\frac{2}{3}}))$
7:
$t_{k+1}=\frac{1+\sqrt{1+4t_{k}^{2}}}{2}$
8:
$\theta_{k+1}=\alpha_{k}+\left(\frac{t_{k}-1}{t_{k+1}}\right)(\alpha_{k}-\alpha_{k-1})$
9: Stopping rule: stop if $\alpha_{k}$ has converged10: Output: sparse signal α


## 3. Numerical Experiments

In this section, we conduct two groups of experiments to evaluate the proposed algorithm. In the first group of experiments, we compare the proposed solution with an MSE-based method to recover simulated sparse signals and image denoising under Gaussian and non-Gaussian noise conditions so as to illustrate the superiority of using negentropy as the measurement error. Further, in the second group of experiments, the effectiveness and practicability of the proposed algorithm are verified by comparing with some robust sparse recovery algorithms to recover simulated sparse signals and real-world MRI images under non-Gaussian noise conditions.

### 3.1. Comparison with MSE-Based Method

(1) Sparse Signal Recovery

In this subsection, the performance of the proposed algorithm in recovering sparse signals from noisy measurements is presented. For the sake of comparison, the results of ISTA are also presented. The parameter settings are summarized in Table 1.

The experiments were performed on a 100-dimension 5-sparsity signal, where the non-zero values had random positions and the values were generated following a uniform distribution in the range [−2,−1]∪[1,2]. The measurement matrix D was generated by randomly drawing value from normal distribution N(0,1). In our experiments, we generate 100 samples of sparse signals and measurement matrices, and the corresponding measurements are obtained from Equation (1). The measurement signals were further corrupted by Gaussian and non-Gaussian noise. In the Gaussian case, the noise is generated from N(0,1) distribution. In the non-Gaussian case, impulse noise is adopted, which is generated by a mixed Gaussian distribution model as suggested in [36]. The reconstruction performance is evaluated by the relative norm error between the reconstructed signal and the original signal, which is defined as

(39) $$\mathrm{Relative}\;\mathrm{norm}\;\mathrm{error}(\alpha_{i})=\frac{||\alpha_{origi}-\alpha_{i}|{|}_{2}}{||\alpha_{origi}|{|}_{2}}$$

**Figure 1:**
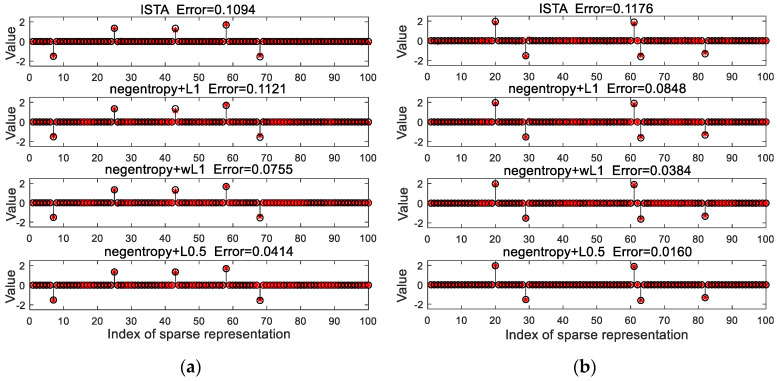
Comparison between original signal (black) and recovered signal (red): (**a**) with Gaussian noise; (**b**) with non-Gaussian noise.

Figure 1 presents the performance of ISTA and our proposed algorithms with 16 dB of Gaussian noise and non-Gaussian noise. As shown in Figure 1a, the relative error of ISTA is slightly smaller than that of the proposed negentropy algorithm with $\ell_{1}$ norm constraint, which indicates that the proposed negentropy objective function has no obvious advantage in recovering signals with Gaussian noise. With non-Gaussian noise, however, it is shown in Figure 1b that the negentropy-based algorithms have higher recovery accuracy, which indicates that the proposed negentropy objective function has a stronger adaptability to non-Gaussian noise. Moreover, when weighted $\ell_{1}$ norm and $\ell_{0.5}$ norm were adopted as sparse constraints, the error further decreases, which implies that $\ell_{p}$ norm as sparse constraint can improve the accuracy of algorithms.

**Figure 2:**
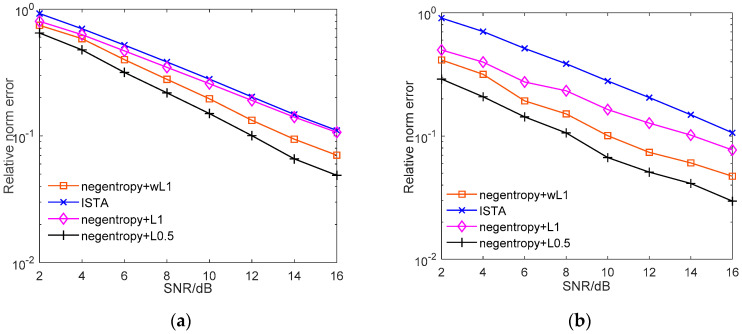
Relative norm error performance versus signal-to-noise ratio (SNR). (**a**) Comparison between ISTA and proposed algorithm with Gaussian noise; (**b**) comparison between ISTA and proposed algorithm with non-Gaussian noise.

Figure 2 presents how the average reconstruction error varies with the signal-to-noise ratio (SNR). The average relative norm error is obtained by averaging over 100 samples of signals and measurement matrices. As Figure 2a shows, the proposed negentropy-based methods have higher reconstruction accuracies under various SNRs. The performance gains further increase when the measurements are contaminated by non-Gaussian noise. As we can see from Figure 2b, the relative error of ISTA is within the level of 10^−1^, but the relative error of the proposed algorithm can reach the scale of 10^−2^, which means that the proposed algorithm model is more robust than conventional algorithms in non-Gaussian noise. Moreover, when weighted $\ell_{1}$ norm or $\ell_{0.5}$ norm were adopted as sparse constraints, the recovery accuracy further increases, and the $\ell_{0.5}$ norm case has superior performance, under which lower reconstruction error can be achieved with high probability.

**Figure 3:**
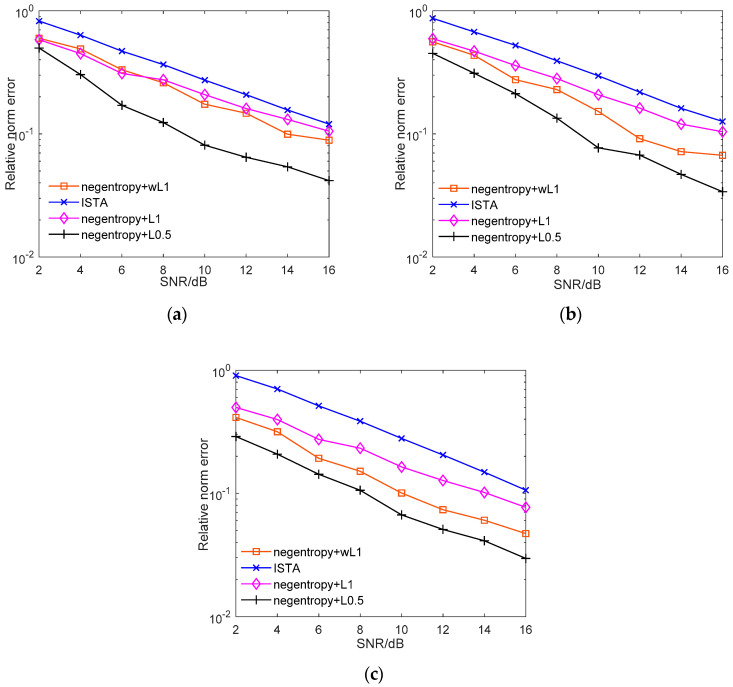
Relative norm error performance versus SNR. (**a**) Comparison between ISTA and proposed algorithm with 40% compression; (**b**) comparison between ISTA and proposed algorithm with 50% compression; (**c**) comparison between ISTA and proposed algorithm with 60% compression.

Figure 3 further illustrates the reconstruction performance with non-Gaussian noisy measurements under various compression ratios. As shown in Figure 3, as the compression ratio decreases, despite the performance of the algorithms deteriorating, the negentropy algorithm always maintains a lower relative error. The $\ell_{0.5}$ norm-based negentropy algorithm presents the best performance, which indicates that the negentropy algorithm can exhibit more stable performance with different compression ratios and also proves that $\ell_{p}$ norm has superior sparse constraint effect to $\ell_{1}$ norm.

**Figure 4:**
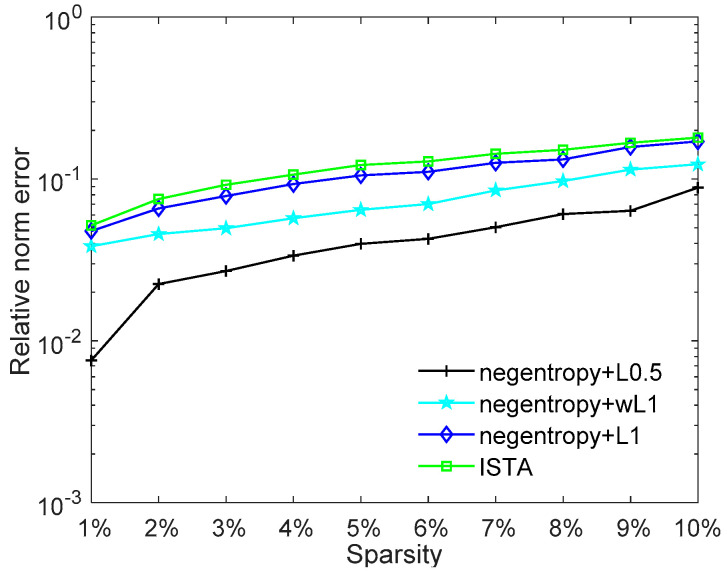
Relative norm error performance versus sparsity.

The impact of sparsity on the reconstruction performance is further presented in Figure 4. As Figure 4 shows, the reconstruction error increases with the sparsity. The negentropy algorithms present better performance than ISTA. The reconstruction error of the negentropy algorithm based on $\ell_{1}$ norm or weighted $\ell_{1}$ norm can only reach the level of 10^−1^ when the signal has low sparsity levels. The negentropy algorithm based on $\ell_{0.5}$ norm has the best performance; the relative error is always kept within 10^−2^ level of magnitude, which shows better suitability for signals with low sparsity.

**Figure 5:**
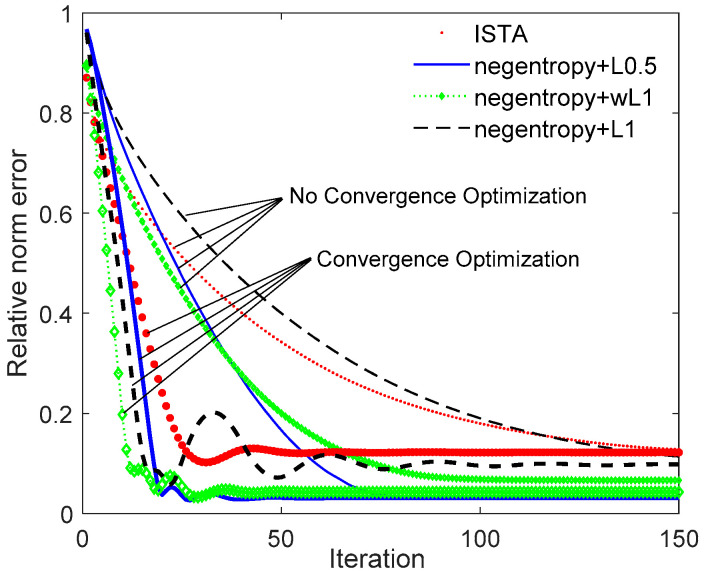
Relative norm error performance versus iterations.

Figure 5 depicts the relative error with iterations. As the number of iterations increases, the relative error will gradually decrease and eventually converge. As we can see from Figure 5, the convergence performance can be significantly improved after convergence optimization. Taking the negentropy algorithm based on $\ell_{0.5}$ norm as an example, the curve tends to be gentle after 75 iterations without accelerating convergence. After the convergence rate is optimized, convergence is achieved in about 40 iterations. Thus, by means of optimizing the convergence rate based on FISTA, the negentropy algorithm also proves stronger convergence is guaranteed compared to ISTA.

(2) Image Denoising Experiments

In order to test the performance of the proposed algorithms in the application, we applied them to image denoising, which performs better compared with ISTA in these experiments. The details of the image denoising experiment are described below. We selected the image named ‘Cameraman’ as the target, which is a 512 × 512-pixel gray-scaled photograph. Gaussian noise and salt and pepper noise were added to the image, which was separated into 12 × 12-pixel small patches with an interval of 2 between patches to form an input set $X\in {\mathbb{R}}^{144\times 63001}$. According to the signal model in Equation (1), we generate a 144 × 256 overcomplete discrete cosine transform (DCT) distributed dictionary for the denoising task. The sparse representation of each patch in the dictionary is recovered by the proposed algorithm and ISTA, which removes the noise in the images. The parameter settings of the algorithms are summarized in Table 2. The peak signal-to-noise ratio (PSNR) of the recovered images and the structural similarity (SSIM) index between the recovered images and original images were used to evaluate the denoising performance.

**Figure 6:**
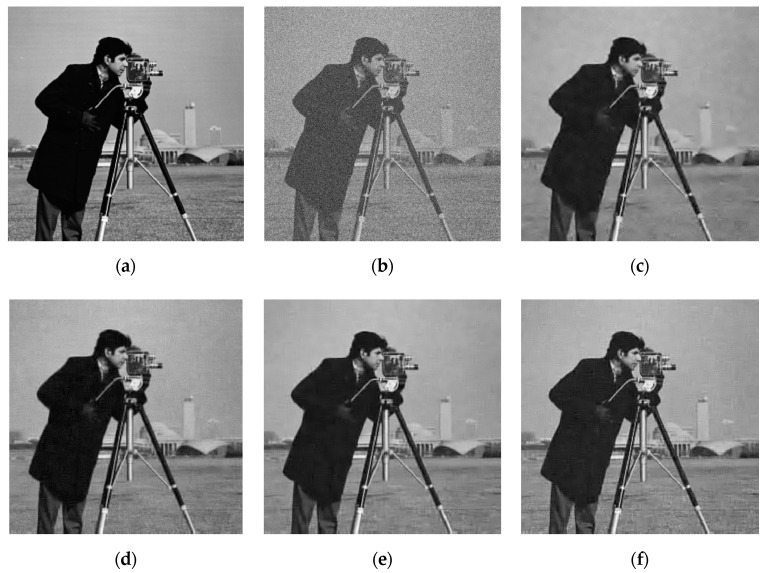
Image denoising result with Gaussian noise: (**a**) original image; (**b**) noised image; (**c**) ISTA; (**d**) negentropy + $\ell_{1}$-norm; (**e**) negentropy + weighted $\ell_{1}$ -norm; (**f**) negentropy + $\ell_{0.5}$ -norm.

Figure 6 shows the results of recovering images with 20.17 dB Gaussian noise using ISTA and the proposed algorithms. By comparing (c) and (d), we can see that the PSNR value of the image recovered by ISTA is 1 dB larger than that of the negentropy algorithm. However, from the perspective of visual effects, the image recovered by the negentropy algorithm based on $\ell_{1}$ norm are relatively clear, and the image recovered by ISTA are somewhat blurred. Therefore, the negentropy algorithm has a certain denoising effect for images with Gaussian noise, but it has no obvious advantage over ISTA.

**Figure 7:**
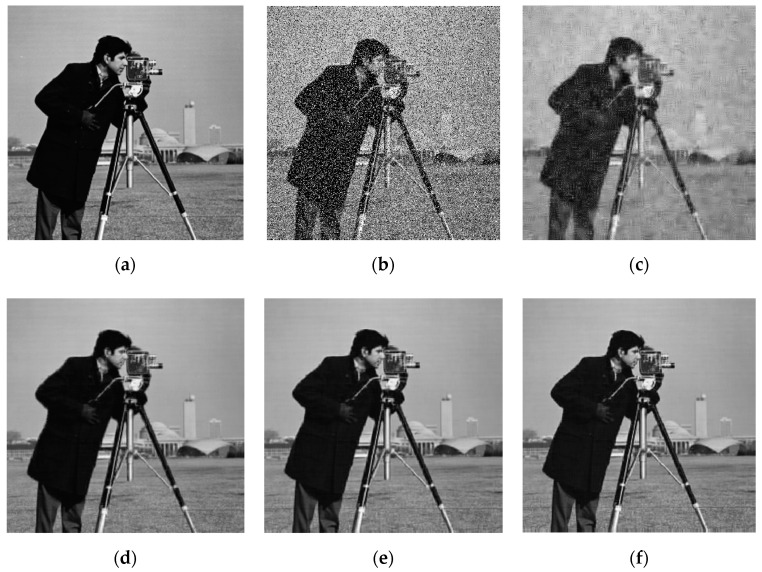
Image denoising result with salt and pepper noise: (**a**) original image; (**b**) noised image; (**c**) ISTA; (**d**) negentropy + $\ell_{1}$-norm; (**e**) negentropy + weighted $\ell_{1}$ -norm; (**f**) negentropy + $\ell_{0.5}$ -norm.

Figure 7 is the results of recovering an image with salt and pepper noise with 30% density using ISTA and the proposed algorithms, which shows that the images recovered using ISTA are very blurred and the PSNR is only 20.24 dB. It can be judged that ISTA can hardly recover the images corrupted by strong salt and pepper noise. The proposed algorithms, however, reconstruct the original image clearly and the PSNR value is over 30 dB, which shows that the algorithm based on negentropy is able to adapt to the influence of non-Gaussian noise. Compared with (d–f), it can be seen that the PSNR value of the image recovered using the negentropy algorithm based on $\ell_{p}$ norm is 1.3 dB higher than that recovered using the negentropy algorithm based on $\ell_{1}$ norm, and the visual effects are better, which demonstrates that $\ell_{p}$ norm, as a sparse constraint, can make the reconstruction accuracy of the algorithm higher and plays a key role in the restoration of image details. For the convenience of comparison, the PSNR results in two noise conditions are shown in Table 3.

**Figure 8:**
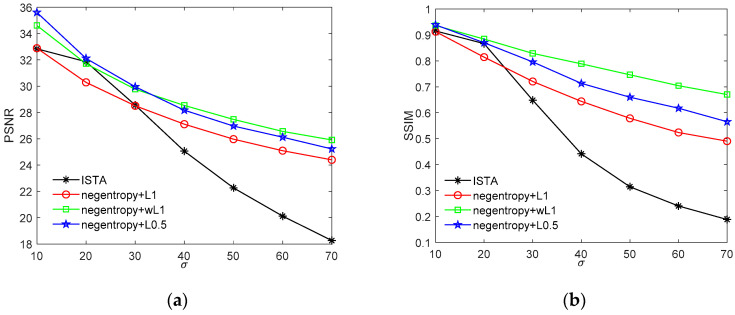
Comparison between ISTA and the proposed algorithm for recovering image with Gaussian noise with standard deviation ranging from 10 to 70. (**a**) PSNR value of recovered image versus noise intensity; (**b**) structural similarity (SSIM) value of recovered image versus noise intensity.

The PSNR and SSIM index values of the denoised images are presented in Figure 8. As we can see, both the PSNR and the SSIM index decrease gradually as the Gaussian noise intensity increases. Despite the fact that the ISTA algorithm presents comparable performance with the proposed algorithms when the standard deviation of the Gaussian noise is around 20, its performance rapidly decreases with the noise intensity. When the Gaussian noise is strong, the proposed algorithm presents much better performance in both PSNR and SSIM, which indicates that the negentropy algorithms have strong adaptability to different noise intensity.

**Figure 9:**
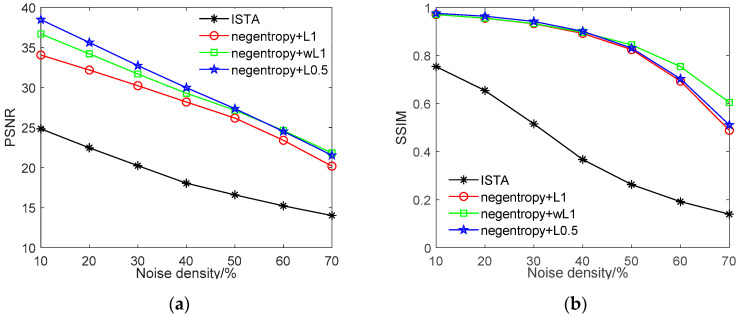
Comparison between ISTA and proposed algorithm for recovering image with salt and pepper noise with density from 10% to 70%. (**a**) PSNR value of recovered image versus noise density; (**b**) SSIM value between recovered images and original images versus noise density.

Figure 9 presents the denoising performance with various densities of salt and pepper noise. As we can observe from Figure 9, the ISTA algorithm can only recover images with salt and pepper noise density below 20%, and the recovered image is extremely blurred, where the maximum SSIM value only reaches 0.74. Furthermore, when the noise density in the image is higher, ISTA is unable to recover a clear image. The negentropy algorithm, on the other hand, can recover the original image with much higher accuracy. For instance, when the noise density in the image is less than 30%, all three negentropy-based methods have PSNR values of more than 30 dB and SSIM values of more than 0.9. The negentropy algorithm with $\ell_{0.5}$ norm achieves the best performance, with PSNR = 38 dB and SSIM = 0.97, when the noise density is 10%. With weighted $\ell_{1}$ norm as constraint, although the PSNR value is lower than $\ell_{0.5}$ norm, the structure similarity between the recovered images and the original images is higher, leading to better visual effects.

### 3.2. Comparison with Robust Methods

(1) Sparse Signal Recovery

In this part, we conduct sparse signal recovery experiments under non-Gaussian noise and use several well-known, robust sparse recovery algorithms for comparison, including Huber-FISTA [20], YALL1 [21] and LqLA-ADMM [22]. The construction of the simulated k-sparse signal is similar to that in Section 3.1. The length of the sparse signal and the number of measurements are set at N = 100, M = 60. An M×N orthonormal Gaussian random matrix is used as the measurement matrix. Impulse noise with SNR = 16 dB. Since the original sparse signal is unknown, we choose $\lambda =\xi {\left\|D^{T}x\right\|}_{\infty }$ (as proposed in [37]) with ξ=0.05 for all algorithms.

**Figure 10:**
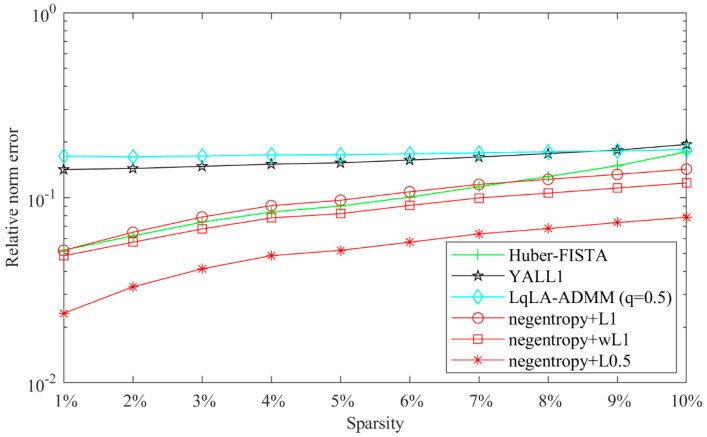
Relative norm error performance versus sparsity.

**Figure 11:**
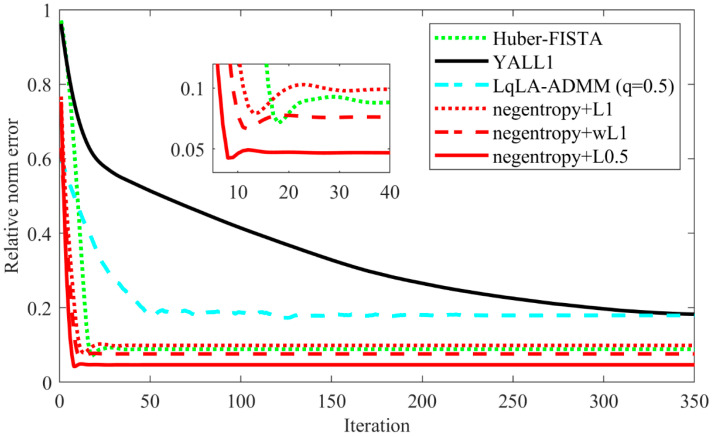
Relative norm error performance versus iterations.

Figure 10 presents the recovery performance of the compared algorithms versus sparsity with k∈[1~10]. All algorithms are conducted 300 times for each k. Figure 11 shows the relative error versus iterations with fixed k = 5. As shown in these figures, the negentropy algorithms (except the $\ell_{1}$ norm-based negentropy algorithm in low sparsity) outperform other compared algorithms in terms of both recovery performance and convergence speed, especially in the case of $\ell_{0.5}$ norm.

(2) Magnetic Resonance Imaging Example

**Figure 12:**
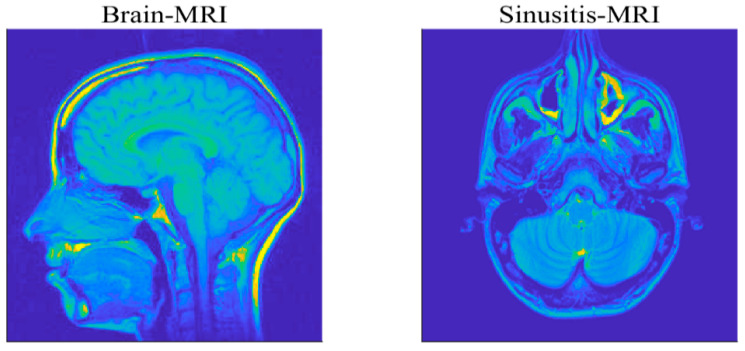
Two test MRI images.

To further verify the effectiveness and practicability of the proposed algorithms, this part will focus on the reconstruction of medical images. The used MRI images are of size 256×256, for a brain MRI and sinusitis MRI, as shown in Figure 12, and evaluate the recovery performance by the value of PSNR. We utilize the Haar wavelets as the basis for sparse representation of the images. The compression ratio *M*/*N* is set as 0.4 and the measurement matrix is a partial DCT matrix. A Gaussian mixture model (GMM) $\left(f=\rho N(0,\sigma^{2})+(1-\rho )N(0,k\sigma^{2})\right)$ is taken to model the impulsive noise, where the parameters ρ∈(0,1) and k>1 respectively control the proportion and the strength of outliers in the noise [19]. To ensure fairness of comparison, the λ in each algorithm is selected by providing the best performance in terms of relative error of recovery [22].

**Figure 13:**
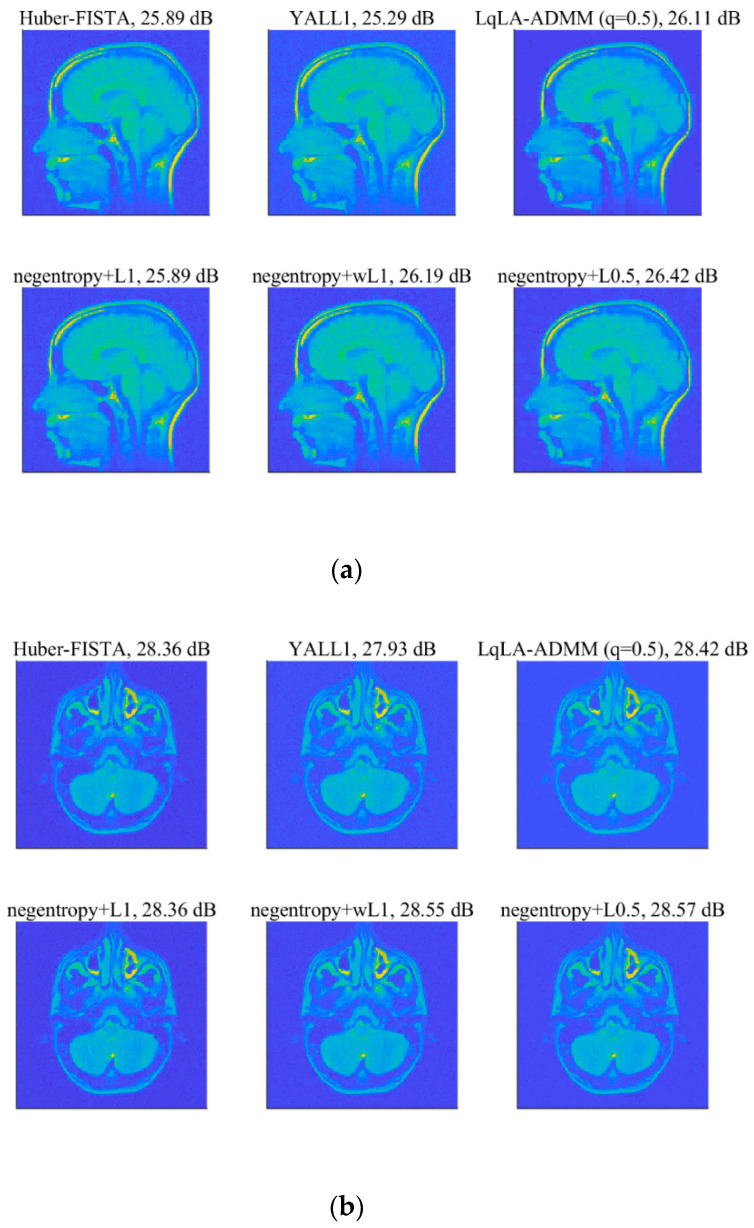
Recovery results of MRI images with the GMM noise: (**a**) brain MRI.; (**b**) sinusitis MRI.

**Figure 14:**
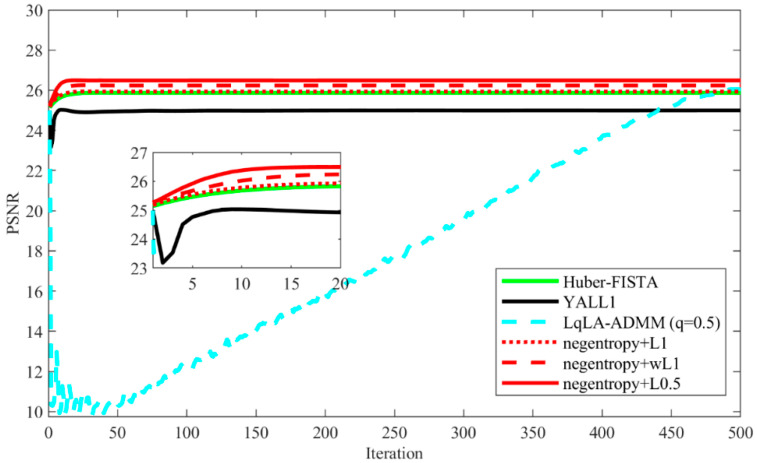
PSNR versus iterations in recovering the brain MRI image.

Figure 13 shows the recovered MRI images of all algorithms under the GMM noise with ρ=0.9,k=1000 and SNR = 20 dB. The PSNR results are shown in Table 4. It can be seen that each algorithm can successfully reconstruct the two MRI images. Quantitatively, it is observed that the PSNR of the MRI images recovered using negentropy algorithms based on $\ell_{p}$ norm have a higher value than those recovered by other compared algorithms and the negentropy algorithm based on $\ell_{0.5}$ norm obtains the best recovery performance. Particularly, for the brain MRI image, the PSNR gains of negentropy + $\ell_{0.5}$ over Huber-FISTA, YALL1 and LqLA-ADMM (q = 0.5) are 0.53 dB, 1.13 dB and 0.31 dB, respectively. Furthermore, Figure 14 presents the convergence curve of PSNR versus iterations in recovering the brain MRI image. It can be seen that compared with LqLA-ADMM (q = 0.5) and Huber-FISTA, the negentropy algorithm requires less iterations to converge. Thus, the proposed algorithm is more efficient for the recovery of MRI images in terms of accuracy and convergence speed.

## 4. Conclusions

In this paper, we propose a sparse signal recovery model based on negentropy maximization. To improve the robustness of the sparse signal recovery algorithm under different types of noise interference, $\ell_{p}$ norm is adopted as the sparsity constraint. We presented two approaches to solve the optimization problem. One is to approximate the $\ell_{p}$ norm to the weighted $\ell_{1}$ norm, which is a convex function and joins the information of the sparse signal from the previous iteration, and then to solve the minimum value using the corresponding approximation operator. The other is to use the approximate operator of IHTA to solve the minimum value of the problem to solve the problem with $\ell_{0.5}$ constraint. To improve the convergence performance, we further optimize the convergence rate of the proposed algorithm based on FISTA to reduce the number of iterations. Numerical experiments were conducted on sparse signal reconstruction, image denoising and MRI images recovery.

In the sparse signal reconstruction task, the proposed algorithms presented better reconstruction performance over ISTA, especially with non-Gaussian noise. Furthermore, the algorithm based on $\ell_{p}$ norm can recover the sparse signal more accurately with higher probability than all the other algorithms. In the image denoising task, the negentropy algorithm has a better denoising effect over the ISTA algorithm when the images are corrupted by non-Gaussian noise, which is more significant when salt and pepper noise is considered. Moreover, the image recovered by the $\ell_{p}$ norm-based algorithm is better than that by the $\ell_{1}$ norm-based algorithm regarding both visual effects and objective evaluation criteria. In the MRI images recovery task, under the circumstance of GMM noise, the proposed negentropy algorithm based on $\ell_{0.5}$ norm can achieve the best recovery performance among the reference algorithms and requires less iterations to converge. In further work, we plan to develop a data-driven approach for adaptively adjusting the parameters of the proposed method and investigate the effectiveness of our approach for wireless sensor networks and the internet of things.

## Figures and Tables

**Table 1:** The parameter setting of algorithms.

Algorithm	Parameters
ISTA	$\lambda =3,\eta =4\times 10^{-3}$
Fast Negentropy + $\ell_{1}$ norm	$\lambda =3,\eta =4\times 10^{-3},c=1$
Fast Negentropy + weighted $\ell_{1}$ norm	$\lambda =3,\eta =4\times 10^{-3},c=1,$ $\delta =1\times 10^{-7},p=0.9$
Fast Negentropy + $\ell_{0.5}$ norm	$\lambda =2,\eta =2.5\times 10^{-3},c=1$

**Table 2:** The parameter setting of algorithms in image denoising experiment.

Algorithm	Parameters
ISTA	$\lambda =33,\eta =5\times 10^{-2}$
Fast iterative Negentropy $\ell_{1}$ norm	λ=1.5,η=0.3,c=1
Fast iterative Negentropy + weighted $\ell_{1}$ norm	λ=0.65,η=0.6,c=1,$\delta =1\times 10^{-7},p=0.9$
Fast iterative Negentropy + $\ell_{0.5}$ norm	λ=2.2,η=0.6,c=1

**Table 3:** Denoising results (peak signal-to-noise ratio (PSNR) in dB) of the different methods in two noise conditions.

Noise Conditions	Noised Image	ISTA	Negentropy + L1	Negentropy + wL1	Negentropy + L0.5
Gaussian noise	20.17	30.34	29.31	30.67	30.86
Salt and pepper noise	10.35	20.24	30.24	31.69	32.74

**Table 4:** Comparison of PSNR (dB) values obtained by different methods under Gaussian mixture model (GMM) noise.

Images	Huber-FISTA	YALL1	LqLA-ADMM (q = 0.5)	Negentropy + L1	Negentropy + wL1	Negentropy + L0.5
Brain MRI	25.89	25.29	26.11	25.89	26.19	26.42
Sinusitis MRI	28.36	27.93	28.42	28.36	28.55	28.57
